# Current development in genetic engineering strategies of *Bacillus* species

**DOI:** 10.1186/1475-2859-13-63

**Published:** 2014-05-03

**Authors:** Huina Dong, Dawei Zhang

**Affiliations:** 1 Tianjin Institute of Industrial Biotechnology, Chinese Academy of Sciences, Tianjin 300308, China; 2 Key Laboratory of Systems Microbial Biotechnology, Chinese Academy of Sciences, Tianjin 300308, China; 3 National Engineering Laboratory for Industrial Enzymes, Tianjin 300308, China

**Keywords:** *Bacillus* species, Genetic engineering strategies, Counter-selection marker, Operator-repressor system, Toxin gene, Site-specific recombination

## Abstract

The complete sequencing and annotation of the genomes of industrially-important *Bacillus* species has enhanced our understanding of their properties, and allowed advances in genetic manipulations in other *Bacillus* species. Post-genomic studies require simple and highly efficient tools to enable genetic manipulation. Here, we summarize the recent progress in genetic engineering strategies for *Bacillus* species. We review the available genetic tools that have been developed in *Bacillus* species, as well as methods developed in other species that may also be applicable in *Bacillus*. Furthermore, we address the limitations and challenges of the existing methods, and discuss the future research prospects in developing novel and useful tools for genetic modification of *Bacillus* species.

## Introduction

*Bacillus subtilis* and some related *Bacillus* species are non-pathogenic, free of exotoxins and endotoxins, and have a recognized history of safe use in foods. These species are also useful for fermentation and large-scale cultivation. Despite these natural advantages, protocols using *Bacillus* species lag far behind those for *Escherichia coli* and *Saccharomyces cerevisiae*, which are the most widely used cellular factories for producing industrially-important enzymes and biochemicals [1,2]. Therefore, efficient genetic manipulation and highly developed systems-level strategies for developing *Bacillus* species as microbial cell factories are needed. Recently, *Bacillus* species have received increased attention for their use in genetic engineering and production of heterologous proteins [3], valuable enzymes, vitamins, platform chemicals, and antibiotics [4-6].

The complete genome sequences of several *B. subtilis* strains have been determined and annotated, stimulating novel methods for interpreting metabolic pathways and supplying an overview of protein machinery [7]. The completion of the genome sequences of industrially-important *Bacillus* species (*Bacillus licheniformis*, GenBank accession number CP000560 and *Bacillus amyloliquefaciens*, GenBank accession number AE017333) revealed a high degree of homology to *B. subtilis*[8-10]. These complete genome sequences not only enhance our understanding of these strains, but also allow advances in genetic manipulations in other *Bacillus* species [6]. With rapid developments in post-genomic studies, simple and efficient genetic tools are required to conveniently enable multiple modifications of the genomes of *Bacillus* species.

Classical chromosomal modification is based on the insertion of a selectable marker, usually a drug resistance gene, into the chromosome of a bacterial strain [11]. Using this strategy, a second selective maker gene is required to introduce another chromosomal modification, so the number of available selection genes limits the feasibility of multiple chromosomal modifications. Moreover, the selectable gene should be removed by single-crossover recombination if the strain is to be used for further genetic manipulation. In addition, the chance of obtaining a positive strain is relatively low, and the selection process is laborious. To overcome these problems, methods that can eliminate marker cassettes in the primary transformants are desperately needed.

Recently, useful tools for genetic modification of *Bacillus* species have emerged from the fields of systems and synthetic biology. Thus, it is necessary to summarize recent progress, current obstacles, and future goals to inspire more research interest and advance studies in this field. First, we summarize the progress in genetic modification strategies of *Bacillus* species, including several kinds of marker-free genetic modification methods and site-specific recombination strategies. Next, we discuss some strategies developed in other species that could also be used in *Bacillus* species. Lastly, we compare current genetic engineering strategies, analyze their challenges and limitations, and discuss future research goals for developing novel and useful tools for genetic modification of *Bacillus* species.

### Operator-repressor system-based genetic engineering strategies

**Figure 1:**
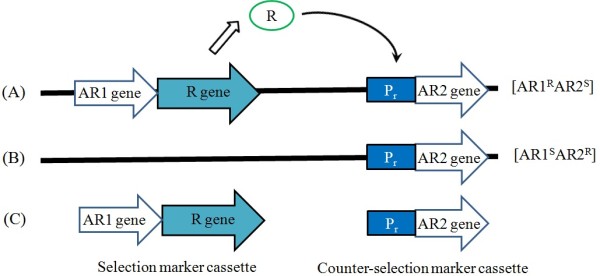
**Operator-repressor system-based genetic engineering strategy. (A)** In the presence of the R gene, the constitutively expressed R protein specifically binds to the P_r_ promoter and represses promoter activity. **(B)** When the R gene is excised from the genome, the P_r_ promoter is activated and mutants can be selected by antibiotic resistance. **(C)** Diagrams of selection and counter-selection marker cassettes. R: Repressor, AR: Antibiotic resistance, P_r_: Promoter. [AR1^R^], [AR1^S^], [AR2^R^], and [AR2^S^] are AR1-resistant, AR1-sensitive, AR2-resistant, and AR2-sensitive bacterial phenotypes, respectively.

The development of various inducible promoter systems has played an important role in the analysis of gene expression and function. Promoters responsive to an assortment of inducing agents, including heavy metals, hormones, and heat shock, as well as several viral, cellular, and bacterial regulatory factors, have been successfully used to manipulate gene expression. Some systems have been applied to genetically engineer *Bacillus* species, and the selection and counter-selection marker cassettes are shown in Figure 1.

#### cI as a counter-selection marker

The CI repressor gene (*c*I*857* or *c*I) from *E. coli* bacteriophage lambda encodes the CI repressor, which can bind to the P_r_ promoter of lambda phage to suppress its promoter activity. Itaya [12] developed a counter-selection method in which a neomycin-resistance gene is regulated by P_r_. This system, controlled by the presence/absence of the CI repressor, allows precise selection for marker-free genetic modification of the chromosome [13-15]. Similarly, Uotsu-Tomita *et al.*[16] developed a double *c*I-P_r_ method for positive selection of *B. subtilis* recombinants, and Tsuge *et al.*[17] obtained markerless deletion of *sfp*, *degQ*, and *ppsABCDE* using the *c*I-P_r_ system. This method can be repeatedly used by changing the location of the *c*I gene [18].

#### araR as a counter-selection marker

*araR* encodes a negative regulator of the *ara* operon that can be induced by L-arabinose in *B. subtilis*[19]. Liu *et al.*[20] developed a method in which the chromosomal *araR* locus was replaced by P_*ara*_-*neo*, which confers neomycin resistance. By adding L-arabinose to the growth medium, the expression of P_*ara*_-*neo* can be induced based on the presence of *araR* in the chromosome. First, the selection marker cassette *cat-araR* is inserted upstream of the target gene via recombination, and then selected for with chloramphenicol. Next, single-crossover recombination between two downstream regions removes the *cat-araR* cassette, together with the target gene. The remaining P_*ara*_-*neo* in the genome can be used again for further genetic modification. The authors obtained 3.8-kb and 41.8-kb deletion strains using this system that left no selective marker gene in the targeted loci.

### Pyrimidine metabolism-based genetic engineering strategies

**Figure 2:**
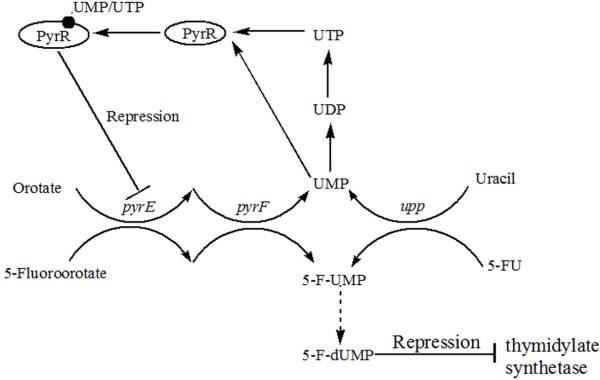
**Summary of pyrimidine metabolism pathway and mechanisms of action of pyrimidine analogs.** Orotate is metabolized to UMP by OPRTase and OMPdecase, encoded by *pyrE* and *pyrF*, respectively. These enzymes also convert 5-fluoroorotate to 5-fluoro-UMP. *pyrR* encodes an mRNA-binding attenuator (PyrR) that negatively regulates *pyr* expression by sensing UMP or UTP. UMP is also produced from uracil by UPRTase, encoded by *upp*. UPRTase converts 5-FU to 5-fluoro-UMP, which is further metabolized to the toxic metabolite 5-fluoro-dUMP. 5-fluoro-dUMP is a strong inhibitor of thymidylate synthetase, which provides the sole *de novo* source of dTMP for DNA biosynthesis.

Common approaches for counter-selection exploit genes involved in purine or pyrimidine metabolism and are based on the fact that purine or pyrimidine analogs can be converted to toxic compounds. Plating cells on media containing the analog leads to a strong selection for clones that have lost the chromosomally-integrated copy of the gene encoding the converting enzyme. Therefore, parental strains used for genome modification must lack the respective gene for purine or pyrimidine nucleotide biosynthesis. Recently, *upp* and *pyrF*, encoding uracil phosphoribosyltransferase (UPRTase) and orotidine 5′-phosphate decarboxylase (OMPdecase), respectively, have been used as counter-selection markers in *Bacillus* species. The relevant pyrimidine metabolism pathway and mechanisms of action of pyrimidine analogs are shown in Figure 2.

#### upp as a counter-selection marker

UPRTase catalyzes the key reaction of the pyrimidine salvage pathway, from uracil to UMP, in many microorganisms. The toxic pyrimidine analog 5-fluorouracil (5-FU) can be converted to 5-fluoro-UMP by UPRTase. The latter compound can be further catalyzed into 5-fluoro-dUMP, which is a strong inhibitor of thymidylate synthetase. Deletion of *upp* endows the mutant strain with resistance to 5-FU.

Fabret *et al.*[21] developed a method using *upp* as a screening marker in *B. subtilis*. A PCR-generated DNA fragment, which consists of the target gene with a desired mutation linked to a *upp* cassette, was inserted into the genome by double-crossover recombination and selected for based on phleomycin resistance. The *upp* gene was excised through recombination of the direct repeats (DR) flanking the *upp* cassette in the linear DNA, and 5-FU-containing medium was used to select for strains that contained the desired chromosomal mutation.

The *upp* cassette has also been used to functionally analyze *B. subtilis* genes by constructing a mutant library [22], point mutations [23], and gene-null strains [24,25]. Morimoto *et al.*[26] generated a genome-reduced *B. subtilis* strain, MGB874, using the *upp* cassette to sequentially knock out genes. Compared with the parental *B. subtilis* 168 genome, the genome of strain MGB874 is depleted by 874 kb (20.7%), including 865 genes.

Tanaka *et al.*[27] developed an improved counter-selection system that combines the *upp* and *c*I-P_r_ systems. In this system, the master strain (MS) TF8A Δ*upp*:: λP_r_-*neo* was constructed by replacing *upp* with a λP_r_-*neo* cassette, the counter-selection marker mentioned above. The *upp-phleo-cI* cassette undergoes homologous replacement with a targeted chromosome region after being introduced into the MS. Positive selection for cassette integration is based on resistance to phleomycin. The *upp* gene and the *sak* promoter construct P_sak_-λ*c*I are used for counter-selection and cassette removal.

Recently, Shi *et al.*[28] developed a method combining *upp* and double-strand break (DSB) repair, which is caused by exogenous endonuclease I-*Sce*I and *comK* overexpression, for fast preparation of competent cells. First, a foreign dsDNA fragment is integrated into the chromosome via double-crossover. The *upp* cassette can then be excised by a second intramolecular homologous recombination. The DSB repair potently induces the second intramolecular recombination, which enhances the frequency of resolution by one to two orders of magnitude.

A method based on *upp* has also been used in *Bacillus* species other than *B. subtilis.* Wemhoff *et al*. [29] developed an *upp*-based deletion method for *Bacillus pumilus* in which master strains used for gene deletion are generated by targeted deletion of a set of genes, including the essential sporulation gene *yqfD*, enabling rapid allelic exchange between *upp* and *hsdR*. The *hsdR* gene encodes the restrictase of a type I restriction modification system, and its deletion makes a strain readily transformable. The resultant *B. pumilus* mutant is easily transformable with plasmid DNA isolated from *E. coli* strains. In addition, direct gene disruption is possible, which enables relatively rapid genetic manipulations.

#### pyrF as a counter-selection marker

Orotate phosphoribosyltransferase (OPRTase) and OMPdecase, encoded by *pyrE* and *pyrF* respectively, are essential enzymes for metabolism of orotic acid to UMP and 5-fluoroorotate to 5-fluoro-UMP. 5-fluoro-UMP can be further converted into the toxic metabolite 5-fluoro-dUMP. The PyrR protein (encoded by *pyrR*) is an mRNA-binding attenuator, which regulates expression of pyrimidine biosynthetic (*pyr*) genes by sensing UMP or UTP.

Suzuki *et al.*[30] established a counter-selection system based on deletion of *pyrR* and *pyrF* in *Geobacillus kaustophilus* HTA426. The disruption of *pyrF* and *pyrR* makes the MS auxotrophic for uracil and resistant to 5-fluoroorotate. Heterologous β-galactosidase and α-amylase genes were integrated in the genome of *G. kaustophilus* by *pyrF*-based counter-selection using pGAM plasmids, without leaving the marker in the target loci. This system may be applied to other organisms harboring *pyrR*, such as *Bacillus*-related bacteria.

### Auxotrophy-based genetic engineering strategies

#### A lysine-auxotrophic strain combined with an operator-repressor system

The *lysA* gene of *B. subtilis* 168 encodes diaminopimelate decarboxylase, which catalyzes the final step in the lysine biosynthetic pathway in which meso-diaminopimelate is converted into lysine. The *lysA* gene is essential for the strain to grow on minimal medium [31,32].

##### A lysine-auxotrophic strain combined with blaI

A conditional auxotrophy-based method for removing selection markers was developed by Brans *et al*. [33]. This method combines the use of *blaI*, a spectinomycin resistance gene, with a conditional lysine-auxotrophic *B. subtilis* strain (BS1541). The *blaI* gene of *B. licheniformis* encodes a cytoplasmic repressor (BlaI) that negatively regulates the expression of β-lactamase (encoded by *blaP*) in the absence of penicillin. The *B. subtilis* P_*lysA*_ promoter was replaced with the *B. licheniformis* P_*blaP*_ promoter to obtain strain BS1541, thus *blaI* can confer lysine auxotrophy to the strain. The *blaI* cassette, containing *blaI* flanked by two DR sequences, was inserted into the genome of strain BS1541 by homologous recombination and selected for with spectinomycin, and the cassette was removed by a single-crossover recombination between the two DRs. This strategy can be used consecutively to further modify the *Bacillus* chromosome.

##### A lysine-auxotrophic strain combined with lacI

A similar method was developed by Zhang *et al.*[34]. A conditionally lysine-auxotrophic *B. subtilis* strain (BS-PS) was generated by substituting the P_*lysA*_ promoter with an isopropyl-β-D-thiogalactopyranoside (IPTG)-inducible P_*spac*_ promoter. A vector containing *lacI*, which encodes a repressor of the P_*spac*_ promoter, and a chloromycetin resistance gene was used to generate specific integrating vectors. The integration of these vectors into the BS-PS chromosome, and the excision of *lacI* and the chloromycetin resistance gene, were both achieved by a single crossover at the homologous arm.

#### A method based on a histidine-auxotrophic strain

*hisF* and *hisI* are the last two genes in the *his* operon of the *B. subtilis* 168 chromosome, and are followed by two genes of unknown function, *yvcA* and *yvcB*. An incomplete histidine biosynthesis operon leads to histidine auxotrophy in *B. subtilis*.

Motejadded *et al.*[35] developed a system based on two plasmids to obtain marker-free recombinant *B. subtilis* strains. One plasmid consists of the 3′-end of *hisF*, the 5′-end of *hisI*, a spectinomycin resistance gene, and a 1.3 kb fragment containing the 3′-end of *yvcA* and the 5′-end of *yvcB*. The other plasmid contains the same *yvc*-region and the 3′- end of *hisF*, but has a complete *hisI* and lacks the antibiotic marker. Spectinomycin-resistant His-auxotrophic mutants are obtained after integration of the first plasmid into the *B. subtilis* genome by double crossover. Insertion of the second plasmid into this mutant leads to a spectinomycin-sensitive His*-*prototrophic strain. These plasmids were successfully applied to integrate the lipase gene of *Bacillus thermocatenulatus* into a *B. subtilis* glucose-regulated system. Morabbi *et al.* also used this system to perform markerless integration of *P*_*mtlR*_*-mtlR-H342D* into the chromosome of *B. subtilis*[36].

### Site-specific recombination-based genetic engineering strategies

**Figure 3:**
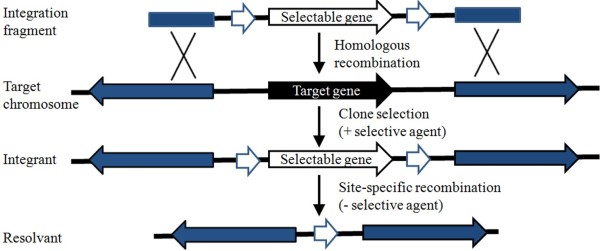
**Site-specific recombination-based genetic engineering strategy.** Selectable genes are often antibiotic resistance genes. White regions represent recognition sites of site-specific recombinases, and blue regions represent sequences homologous between the genome and the integration cassette.

Site-specific recombination (SSR) systems use recombinases that catalyze recombination between two site-specific recognition sites, which generates a desired DNA integration, deletion, or inversion. SSR systems that are used in bacterial genome engineering include Cre/*loxP* from bacteriophage P1 [37], Xis/*attP* from bacteriophage λ [38], and FLP/*FRT*[39] from *S. cerevisiae*. The recombination efficiency of SSR systems is much higher than that of native recombination systems, which makes them applicable for undertaking multiple sequential mutations of the same chromosome (Figure 3). Recently, Cre/*loxP* and Xer/*dif* systems have been used in *B. subtilis* species*.*

#### The Cre/lox system

The Cre/*lox* system is a powerful genetic tool [40] that is widely used in eukaryotic and prokaryotic cells and consists of a Cre recombinase and a pair of *loxP* sites. Cre recombinase catalyzes the reciprocal site-specific recombination between the two *loxP* sites, which does not require any host cofactors or accessory proteins. A pair of modified lox sites, *lox71* (L) and *lox66* (R), are usually used [41] to minimize genetic instability, as microbial genomes can contain multiple native *loxP* sites that could be identified by Cre. A double mutant *lox72* remnant site, which has weaker binding affinity for Cre, is obtained following recombination of *lox71* and *lox66*, allowing for repeated mutations in a single genetic background [42,43].

Yan *et al*. [11] developed a genome engineering procedure for *B. subtilis* by combining the mutant Cre/*lox* system with a fusion PCR method. After the integration of a fusion PCR product (*lox71-spc/zeo-lox66* cassette) into the genome, a thermosensitive vector containing *cre* is introduced to promote recombination between *lox66* and *lox71* sites. The PCR fusion product is only about 1.5 kb long, reducing the occurrence of PCR errors, which in turn reduces the probability of introducing errors into the chromosome. Three mutations have been successfully integrated into the same background strain using this method. Chromosomally-encoded *tetR* in *B. subtilis* was disrupted by insertion of a *lox66*-*aphAIII-lox71* kanamycin resistance cassette, and subsequent marker excision by Cre recombinase led to the assembly of a novel *tetR* allele [44]. Phosphotransacetylase, encoded by *pta*, was ablated using the Cre/*lox* system in a strain of *B. subtilis* 168 engineered to produce L-malate [45]. Finally, five adjacent genes, *nagP*, *gamP*, *gamA*, *nagA*, *nagB*, were also deleted using the Cre/*lox* strategy and a PCR-based chromosome modification method for production of *N*-acetylglucosamine in *B. subtilis*[46].

*Bacillus coagulans* is a promising species for producing bulk chemicals from renewable resources in the industrial field, but there are few genetic tools for its modification. Kovacs *et al.* described a Cre-*lox* system that uses two plasmids for targeted gene modification in *B. coagulans*[47]. pMH77 is an integration vector that carries a *lox66-cat-lox71* cassette flanked by restriction sites that can be used for cloning homologous regions. pMH66 is a Cre-recombinase plasmid that is used to promote recombination between the two *lox* sites. The authors used this technique to develop a LacZ reporter assay for measuring gene transcription and to express heterologous D-lactate dehydrogenase.

Although Cre-mediated recombination and excision of the chromosomal sequence between two *lox* sites is efficient, it does not occur in all cells. To address this, Wang *et al.*[48] developed a procedure combining Cre recombination and the hen egg white lysozyme gene (*hewl*) as a counter-selectable marker that eliminates the cells carrying the selection cassette. This procedure is based on the beta protein of lambda phage, a single-stranded annealing protein, and employs a single-stranded PCR product containing a *lox71-ble-hewl-*P_R_-*lox66* cassette to modify a specific gene. The single-stranded DNA can be protected from exonuclease digestion by beta protein, and can be recombined via beta recombinase-catalyzed annealing at the replication fork [39,49]. The beta protein, regulated by promoter P_RM_ in the lambda cI857 P_RM_–P_R_ promoter system on the thermosensitive plasmid pWY121, promotes homologous recombination. The *hewl* gene is placed after promoter P_R_, which is effective in *B. subtilis*, and is precisely regulated by the CI857 repressor protein [50]. The efficiency of in-frame deletion using this method can reach 100%. As hen egg white lysozyme is active against *Bacillus* species, and its encoding gene is distantly related to *Bacillus* genes [51], it could also be effective in other *Bacillus* species.

Later, Enyeart *et al.*[52] combined retargetable mobile group II introns, or “targetrons”, and the Cre/*lox* system into a versatile platform known as Genome Editing via Targetrons and Recombinases (GETR). Targetrons can be inserted into desired DNA sites with such high efficiency that the inclusion of a selectable marker is not necessary. The *lox* sites are delivered to specific genomic loci by introns, which enable genomic manipulations, and the added flexibility of RNA hairpins formed by the *lox* sites enhances the efficiency. GETR is an efficient bacterial genetic engineering approach with broad host-applicability that can be used to generate insertions, deletions, inversions, and one-step cut-and-paste operations.

#### The Xer/dif system

Chromosome dimers, which are formed during the bacterial life cycle, must be resolved by the bacterial cell machinery for efficient chromosome segregation. The Xer/*dif* site-specific recombination system used by most bacteria resolves these chromosome dimers into monomers using two tyrosine recombinases to perform the recombination reaction at the *dif* site, which consists of 28–30 bp. Xer recombinases are represented by XerC and XerD in Gram-negative bacteria such as *E. coli*[53], and by CodV and RipX in *B. subtilis* and other Gram-positive bacteria [54]. Intramolecular Xer recombination can excise a *dif*-flanked DNA sequence from a chromosomally inserted cassette.

Bloor *et al.*[55] developed a simple and effective method for genetic modification in bacterial chromosomes based on the Xer/*dif* system. The insertion cassette used in this method consists of a selectable marker gene flanked by *dif* sites and homology arms that are homologous to the genomic target. After the insertion cassette integrates into the corresponding chromosomal region via double-crossover recombination, Xer recombinases can integrate the two *dif* sites into one site, thus removing the selectable marker gene. This method eliminates the requirement for an exogenous SSR system, as Xer recombinases are naturally present in bacteria. Furthermore, a counter-selectable gene is not necessary, as the frequency of Xer recombination is sufficient to allow detection of recombinant clones without antibiotic selection. It is worth noting that introducing multiple *dif* sites in close proximity can induce deletions of the intervening gene segments, so this method may not be suitable for modifying multiple adjacent genes. Pohl *et al.*[56] used the Xer system to generate *B. subtilis* mutants with precise deletions in ten extracytoplasmic proteases that affect recombinant protein secretion.

### Toxin gene-based genetic engineering strategies

**Figure 4:**
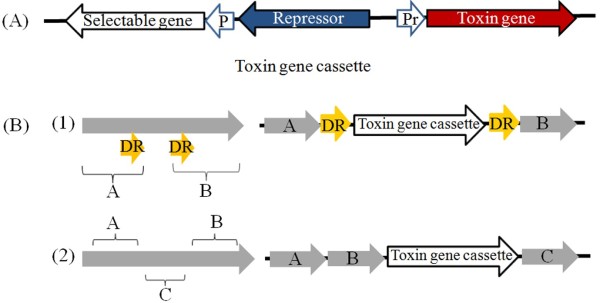
**Toxin gene-based genetic engineering strategies. (A)** Schematic representation of the toxin gene cassette. P: Commonly-used constitutive promoter. Pr: Promoter of operator-repressor system, which is repressed by the repressor and activated by an inducer e.g. P_*spac*_, P_*xyl*_*.* The repressor gene in the cassette can also be deleted in some methods. **(B)** Two different strategies based on toxin gene cassettes. **A**: upstream sequence; **B**: downstream sequence; **C**: sequence for integration of the toxin gene cassette, in combination with A in (2); DR: direct repeat sequence. After integration of the toxin gene cassette into a target chromosome locus via double-crossover recombination [A and B in (1) or A and C in (2)] and positive selection for antibiotic resistance, the cassette is removed by a single crossover event between two DR sequences in (1) or B sequences in (2).

Toxin-antitoxin (TA) systems usually include a functional element consisting of a biologically active protein molecule and its corresponding inhibitor. TA loci are divided into three different types, but the major part of this monograph is devoted to describing type II loci because of the large numbers of known evolutionarily-independent type II gene families. *mazF* and *ccdB* are two well-described toxin genes in the *mazEF* and *ccdAB* type II TA systems, respectively, both of which have been applied in genetic modification systems. The toxin gene cassettes and commonly used genetic engineering strategies based on them are shown in Figure 4.

#### mazF as a counter-selection marker

*mazEF* is one of the best-characterized TA systems. The MazF toxin of *E. coli* is an mRNA interferase that cleaves cellular free mRNAs specifically at ACA trinucleotides [57] to block protein synthesis, which inhibits growth. MazF expression in both bacterial and mammalian cells induces programmed cell death [58,59].

Zhang *et al.* presented a simple method, using *E. coli mazF* as a counter-selection marker, that can be applied to many *Bacillus* species without prior genetic modification of the host [60]. This method uses a *mazF* cassette containing *mazF* under the control of IPTG-inducible P_*spac*_ promoter, a spectinomycin resistance gene, and two flanking DR sequences. After the *mazF* cassette from the linearized delivery vector is integrated into a target chromosome locus via double-crossover recombination, the *mazF* cassette is removed by a single-crossover event between the two DR sequences. This method requires cloning and takes approximately 2 weeks. In addition, *mazF* is regulated by the P_*spac*_ promoter, which has a low induction rate and can have leaky expression in the absence of an inducer in *B. subtilis*[61-63]. Such leaky expression of *mazF* increases the frequency of spontaneous *mazF*-resistant mutants, which decreases the likelihood of isolating colonies with the designed mutation.

Morimoto *et al.*[64] combined the IPTG-inducible expression system with a high-fidelity fusion PCR method to generate marker-free deletion mutants of *B. subtilis.* In this method, sequences were designed for integration and excision of the *mazF* cassette by double- and single-crossover, respectively. This procedure is quicker than the method developed by Zhang *et al.*[60]. However, it still has some limitations, including possible DNA mutations introduced by the 4.0-kb PCR fusion fragment, the difficulty of assembling different DNA fragments, and some leakiness.

Yu *et al.*[65] replaced the IPTG-inducible *spac* expression system with the *xyl* expression system from *Bacillus megaterium*, which has tighter transcriptional regulation and a higher induction rate than the *spac* expression system [62,66]. In this system, *mazF* is placed under the control of the P_*xyl*_ promoter, which is repressed by the xylose-responsive repressor XylR in the absence of xylose. However, the long PCR-fusion fragment-generated mutations and spontaneously-generated *mazF*-resistant mutants should also be taken into consideration when using the above method.

Like its counterpart in *B. megaterium*, the inducible P_*xyl*_ promoter of *B. subtilis* also has strict transcriptional regulation [67] and, in fact, P_*xyl*_ from *B. subtilis* W23 shows tighter regulation than P_*xyl*_ from *B. megaterium*[61,62]. Lin *et al.*[68] constructed a mini-*mazF* cassette containing P_*xyl*_ from *B. subtilis*, *mazF*, and a zeocin resistance gene. The mini-*mazF* cassette is about 2 kb long, which can somewhat reduce the possibility of PCR-induced mutations. The transformation frequency of this cassette is three-fold higher than the above-mentioned *mazF* cassettes, and the rate of spontaneous *mazF*-resistant mutants is low.

#### ccdB as a counter-selection marker

*ccdAB* is often used for positive selection of transformants, primarily in *E. coli* strains. Commercially-available systems (e.g. StabyCloning™ and StabyExpress™, Delphi Genetics SA, Charleroi, Belgium) are based on CcdB toxicity against gyrase and allow one-step selection of transformants, ensuring stable vector plasmid maintenance.

Recently, the plasmid F toxin gene *ccdB* was used as a counter-selection marker to construct markerless mutants of *Vibrio splendidus*[69]. A suicide vector carrying *ccdB* under the control of the arabinose P_*BAD*_ promoter, which can be transferred to any *Vibrio* strain by RP4-based conjugation, was developed. The genetic modification system based on this suicide vector requires a two-step allelic exchange procedure. In the presence of arabinose, the counter-selection provided by the integrated vector enabled efficient markerless gene replacement in both *V. splendidus* and *Vibrio cholerae*.

Although *ccdB* has been used as a positive selection marker for a long time, its use for counter-selection in markerless genetic engineering is limited to a few strains and it has not yet been used in *Bacillus* species*.* Further analysis of *ccdB* and toxin genes from other TA systems is needed to determine whether they may be widely applicable for counter-selection in other species.

### Thermosensitive plasmid-based genetic engineering strategies

Zakataeva *et al.*[70] developed a simple method based on a thermosensitive replication plasmid to introduce markerless mutations into the chromosomes of *B. amyloliquefaciens*. In this method, a delivery plasmid is efficiently introduced into cells for gene replacement, and a two-step replacement procedure mediated by single-crossover events is used. The procedure is efficient and fast and no counter-selection marker or special strain is required. Although this method is designed for *B. amyloliquefaciens*, it has also been successfully adapted to *B. subtilis*. Using this method, Sheremet *et al*. constructed a series of markerless *B. amyloliquefaciens* strains to produce inosine and 5-aminoimidazole-4- carboxamide ribonucleoside [71].

### Transconjugation-based genetic engineering strategies

In some applications, *B. licheniformis* and *B. megaterium* outperform the better-studied microbiological model, *B. subtilis*. However, commonly-used methods for genetic modification of these strains, such as protoplast transformation, are time-consuming and complicated. Recently, some easy markerless deletion methods based on transconjugation have been developed.

#### A sacB-based transconjugation system for B. megaterium

B. megaterium is an industrially-important species, as it has been used to produce heterologous proteins and valuable enzymes. However, genetic manipulation of B. megaterium is difficult, primarily because of low transformation efficiency. Richhardt *et al.*[72] developed a simple and efficient transconjugation method for *B. megaterium*, combining a known transconjugation method [73] and *B. subtilis sacB*, which encodes levansucrase. The activity of this enzyme in the presence of sucrose is lethal to *E. coli*[74], so it is used as a counter-selection marker to eliminate the *E. coli* donor cells after mating. The transfer efficiency of this method is approximately 5 × 10^−5^ transconjugants/recipient, which is sufficient to allow direct selection of mutants in a one-step procedure.

#### A transconjugative plasmid-system in B. licheniformis

Rachinger *et al.*[75] established a markerless gene modification method for *Bacilli* species without natural competence, such as *B. licheniformis*. Chromosomal gene deletion is accomplished by the pKVM series of conjugative shuttle vectors, which contain regions flanking the target gene. These shuttle vectors carry the temperature-sensitive origin of replication from pE194ts and a thermostable β-galactosidase, allowing blue/white screening of recombinant clones on X-gal-containing agar plates, and can be conjugated to *B. licheniformis* and *B. subtilis* strains. Integration of the vector at the target locus, and its subsequent excision, are both mediated by homologous recombination and identified based on appropriate selection markers. These pKVM vectors can be used to efficiently generate deletions and insertions in *B. licheniformis* and other *Bacillus* strains.

### Proposed methods for genetic engineering of Bacillus species

#### Tetracycline-dependent conditional gene knockout in B. subtilis

The tetracycline repressor (TetR) and its reverse mutant (revTetR) can be used for reversible, tetracycline-dependent induction and silencing of gene expression, respectively. Kamionka *et al.*[76] used both of these approaches in *B. subtilis*, as an example of a Gram-positive bacteria. In this system, the genomic *spoVG-lacZ* fusion gene is regulated by one or two *tet* operators, and either TetR or revTetR is controlled by different promoters, allowing precise adjustment of regulatory windows. TetR or revTetR turn expression on or off, respectively, when anhydrotetracycline is added, which means these two components can be used to construct conditional knockouts in *B. subtilis* and many other Gram-positive bacteria.

#### oroP from Lactococcus lactis as a counter-selection marker

The orotate transporter of *L. lactis*, encoded by *oroP*, mediates 5-fluoroorotate sensitivity in *B. subtilis* 168, *E. coli* XL1-Blue, and 5-fluoroorotate-sensitive lactococci. *oroP* is necessary for pyrimidine-auxotrophic derivative strains to use orotate as a sole pyrimidine source [77].

Solem *et al.*[78] developed a selection/counter-selection vector, pCS1966, which harbors *oroP* and can only replicate in *E. coli.* This plasmid can be used for homologous recombination at a specific site, and for integration at bacteriophage attachment sites. The plasmid contains an erythromycin-resistance gene for positive selection of cassette integration, and orotate utilization can be used for counter-selection and cassette excision in a pyrimidine auxotrophic mutant. As *oroP* can be functionally expressed in *B. subtilis*, its use for counter-selection can potentially be exploited in this species.

#### bgl/lacZ as counter-selection markers

β-glucosidase, encoded by *bgl*, can cleave 5-bromo-4-chloro-3-indolyl (BCI) to produce an indoxyl derivative that is toxic to bacteria. Angelov *et al.*[79] described a markerless mutation method that uses *bgl* and *lacZ* as counter-selection markers, and demonstrated the method in the thermophile *Thermus thermophilus* HB27 and in *Micrococcus luteus* ATCC 27141. This method uses a delivery plasmid containing the counter-selection markers and flanking regions of the target gene for efficient gene replacement in a two-step replacement process mediated by single crossover events. As *Bacillus* species are also sensitive to BCI substrate cleavage, this approach could be used to generate markerless chromosomal mutations in *Bacillus.*

### Strategy comparison and prospects

**Figure 5:**
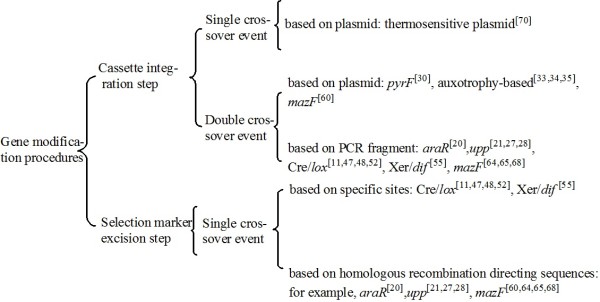
Classification of genetic modification strategies according to different procedures.

Genome engineering strategies usually require two steps: the integration of a disruption cassette into the genome, and the excision of a selectable marker. Examples of these gene modification strategies, classified based on the different procedures used, are shown in Figure 5. Plasmid-borne disruption cassettes can integrate into the genome by single- or double-crossover, whereas those in PCR fragments usually integrate via the latter mechanism. Restriction endonuclease/ligase-dependent methods are not compatible with large scale approaches, and it generally takes about 2 weeks to complete a marker-free modification. In these methods, single-crossover events have a higher rate of positive recombinants in the cassette integration step than in double-crossover events, although the number of false-positive recombinants will increase in the selection marker excision step. In comparison, methods that use fusion PCR or long-flanking homology PCR techniques to generate the disruption cassette can modify a target gene more rapidly; however, these methods are prone to point mutations and may have difficulties in assembling different DNA fragments. High-fidelity PCR would reduce the incidence of point mutations in short fragments (<4 kb) [80].

Marker excision is usually mediated by a single-crossover event, and the efficiency of this step largely determines the overall success of the genetic modification process. SSR can be used to efficiently eliminate the selection marker from the mutated locus, but it leaves remnant sequences (scars) at the targeted site. Some methods using counter-selection markers are scarless, but they are less efficient than methods based on SSR. Thus, methods combining SSR with counter-selection markers have emerged.

Many methods, such as those derived from *upp* deletion, can only be used in strains that have a specific gene mutated, which limits their application. Hence, methods that require no such prior modifications in the host and can be applied to some *Bacillus* species to satisfy the strong demand for a universal unmarked delivery system will receive much attention.

In general, an innovative method to modify genomes of *Bacillus* species should have certain characteristics: 1) it allows markerless or scarless genome manipulation that is both efficient and precise; 2) it has no requirement for any prior mutation prior to genetic modification; 3) it can be used for system-level genetic modifications or can generate multiple genomic mutations simultaneously.

Based on the above analysis, efforts to improve genetic modification technology for *Bacillus* species should focus on: 1) optimizing existing homologous-recombination-based genetic modification methods; 2) introducing more advanced technologies from other species into *Bacillus* species; 3) developing new combinatorial engineering tools for genome-wide modification, such as global transcription machinery engineering [81], tractable multiplex recombineering [82], and multiplex automated genome engineering [83]; 4) adapting novel genome editing engineering technology, such as clustered regularly interspaced short palindromic repeats (CRISPR)/CRISPR-associated sequences and similar multi-loci editing systems, to *Bacillus* species [84,85].

## Conclusions

*Bacillus* species have become important platforms for producing various enzymes and chemicals. A vast range of cellular phenotypes can be obtained in *Bacillus* species by regulating and modifying the corresponding metabolic pathways at global and gene-specific levels. Advances in genetic engineering strategies have helped realize the potential of *Bacillus* species as production hosts for manufacturing commodities, and make *Bacillus* species competitive with the traditional industrial microbes *E. coli* and *S. cerevisiae*.

Many useful tools for genetic modification of *Bacillus* species have been developed in recent years. In this review, we summarized and compared the design principles of current genetic engineering strategies and their recent progress. These strategies still have their own challenges and limitations, so comprehensive and efficient tools for systems-level genetic modifications are still required. We also detailed future research prospects for developing novel genetic modification systems for *Bacillus* species, which are expected to inspire further interest and advance studies in related fields.

## Abbreviations

UPRTase: Uracil-phosphoribosyltransferase; 5-FU: 5-fluorouracil; DR: Direct repeats; MS: Master strain; DSB: Double-strand break; OPRTase: Orotate phosphoribosyltransferase; OMPdecase: Orotidine 5′-phosphate decarboxylase; IPTG: Isopropyl-β-Dthiogalactopyranoside; SSR: Site-specific recombination; GETR: Genome Editing via Targetrons and Recombinases; TA: Toxin-antitoxin; TetR: Tetracycline repressor; BCI: 5-bromo-4-chloro-3-indolyl; CRISPR: Clustered regularly interspaced short palindromic repeats.

## Competing interests

The authors declare that they have no competing interests.

## Authors’ contributions

All authors defined the topic of the review and wrote, read and approved the manuscript. Both authors read and approved the final manuscript.
